# ASSOCIATION OF MANAGED CARE ORGANIZATION HCBS QUALITY AND HEALTH CARE UTILIZATION

**DOI:** 10.1093/geroni/igad104.0809

**Published:** 2023-12-21

**Authors:** Eric Jutkowitz, John Mulcahy, Peter Huckfeldt, Christopher Frenier, Tetyana Shippee

**Affiliations:** Brown University, Providence, Rhode Island, United States; University of Minnesota, Minneapolis, Minnesota, United States; University of Minnesota School of Public Health, Minneapolis, Minnesota, United States; Yale, New Haven, Connecticut, United States; University of Minnesota, Minneapolis, Minnesota, United States

## Abstract

Providing high quality home and community-based services (HCBS) to older adults is a priority, but surprisingly little is known about the association between HCBS quality and client health care utilization. We linked data from the 2018 wave of the National Core Indicators-Aging and Disabilities (NCI-AD) Adult Consumer Survey for participants in the State of Minnesota enrolled in the Elderly Waiver (n=1,449) to their Centers for Medicare and Medicaid Services (i.e., claims data). We attributed respondents in our sample to 1 of 8 managed care organizations (MCOs) responsible for managing the health and HCBS of beneficiaries. For each MCO, we calculated overall plan level HCBS quality using a validated approach based on items in NCI-AD with subdomains of care experience, security and inclusion. Plan quality was the independent variable in a series of multivariate regressions that controlled for county and beneficiary characteristics. Outcomes (yes/no for any utilization in year) included: 1) hospitalizations, 2) emergency department, 3) home health, 4) durable medical equipment, 5) personal care assistance, 6) transportation, 7) case management, 8) home services, and 9) adult day. Quality was positively associated with case management utilization, but no other measure. As the market share of the highest quality plan in a county increased, all beneficiaries (regardless of MCO) in the county used more durable medical equipment, case management services, and adult day care. In conclusion, MCO quality may be driven by client encounters with plans via case managers. A next step is to determine the causal relationship between quality and outcomes.

